# Dysfunction of goal‐directed control in patients with depression and nonsuicidal self‐injury

**DOI:** 10.1002/brb3.2607

**Published:** 2022-05-19

**Authors:** Qi Chen, Meng Liu, Rongzhen Wen, Chuanyong Xu, Zhen Wei, Wei Zhang, Carol A. Seger, Ziwen Peng

**Affiliations:** ^1^ Center for the Study of Applied Psychology, Guangdong Key Laboratory of Mental Health and Cognitive Science and School of Psychology South China Normal University Guangzhou China; ^2^ Department of Child Psychiatry and Rehabilitation, Affiliated Shenzhen Maternity & Child Healthcare Hospital Southern Medical University Shenzhen China; ^3^ Department of Psychology Colorado State University Fort Collins Colorado USA; ^4^ Department of Child Psychiatry, Shenzhen Kangning Hospital Shenzhen University School of Medicine Shenzhen China

**Keywords:** goal‐directed control, nonsuicidal self‐injury, Pavlovian conditioned stimuli, Pavlovian‐to‐Instrumental Transfer

## Abstract

**Background:**

Non‐suicidal self‐injury (NSSI) is a common problem associated with dangerous outcomes. Dysfunction of goal‐directed behavioral control may contribute to NSSI. To test this, we used a novel experimental paradigm (Pavlovian‐to‐Instrumental Transfer, PIT) to test whether patients with NSSI utilize Pavlovian conditioned stimuli (CSs) during goal‐directed control of ongoing behavior.

**Methods:**

Thirty‐five depressed patients with NSSI (D‐NSSI) and thirty‐four healthy controls performed a PIT task. We measured the influence of positive and negative background CSs on instrumental responses for rewards.

**Results:**

The results showed that D‐NSSI performed significantly lower PIT than controls, and PIT measures were negatively correlated with NSSI frequency. Furthermore, in a subset of patients exhibiting high levels of compulsivity, PIT positively moderated the relationship between compulsivity and NSSI frequency.

**Conclusions:**

These results indicate that D‐NSSI patients have difficulties in using different CSs to control ongoing behavior in a goal‐directed manner, and the dysfunction of goal‐directed control may contribute to NSSI.

## Graphical abstract

1. Pavlovian‐to‐Instrumental Transfer (PIT) as a measure of goal‐directed behavior.

2. Patients with depression and nonsuicidal self‐injury (D‐NSSI) showed significantly lower PIT than controls.

3. PIT measures were negatively correlated with NSSI frequency.

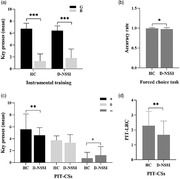

## 1 INTRODUCTION

Nonsuicidal self‐injury (NSSI) is defined as deliberately injuring one's own body tissue by approaches such as burning, cutting, or head‐banging but without the intent to suicide (Nock & Favazza, 2009). NSSI is common: a review of studies published between 1993 and 2012 found a pooled lifetime prevalence of NSSI of 17.2% in adolescents (age 10–17), 13.4% in young adults (age 18–24), and 5.5% in adults (age ≥25 years) (Swannell et al., 2014). NSSI is a robust predictive factor of suicide (Hamza et al., 2012) and the co‐occurrence rate of NSSI and suicide attempts in adolescents engaged in recent NSSI ranges up to 70% (Nock et al., 2006). NSSI can lead to physical harm, heightened aversive feelings and emotions, academic difficulty for students, and decreased overall functioning (Bentley et al., 2014). Given NSSI is so common and dangerous, it is critical to understand the underlying mechanisms of NSSI to aid in prevention and treatment.

Several neural and psychological mechanisms have been proposed to underlie NSSI. These include reinforcement sensitivity, compulsivity, and dysfunction of goal‐directed control. First, several theories of NSSI focused on how NSSI relates to reinforcement. For example, in the four‐function model, NSSI was posited to be maintained through four distinct reinforcement processes depicted by two dichotomous dimensions: positive versus negative and intrapersonal versus interpersonal (Bentley et al., 2014; Nock, 2010). Neuroimaging found that patients with NSSI showed heightened neural sensitivity in reward associated brain regions in response to a monetary reward, suggesting abnormal reinforcement sensitivity in NSSI (Poon et al., 2019; Vega et al., 2018).

Second, NSSI may be characterized by the trans‐diagnostic parameter of compulsivity. Compulsivity is a key feature of many disorders including addictive disorders and obsessive‐compulsive and related disorders (van den Heuvel et al., 2016) and is defined as “the performance of repetitive and functionally impairing overt or covert behavior without adaptive function, performed in a habitual or stereotyped fashion, either according to rigid rules or as a means of avoiding perceived negative consequences” (Fineberg et al., 2014). Previous studies suggest that NSSI can be conceptualized as a form of addiction because its neurobiological (e.g., opioid systems) and psychological (e.g., the aversive withdrawal symptoms) mechanisms are similar to those implicated in addiction (Blasco‐Fontecilla et al., 2016). One study, based on a large sample of 7839 people, suggested that Obsessive‐Compulsive Personality Disorder trait scores can predict NSSI (Bowen et al., 2018). Other studies found that individuals who engage in NSSI have orbital frontal cortex (OFC) and putamen activation abnormities during reward processing (i.e., in gambling tasks), and both of these brain regions are also associated with compulsivity (Osuch et al., 2014; Poon et al., 2019; Vega et al., 2018).

A critical mechanism underlying compulsivity is reduction in goal‐directed control of behavior and overreliance on habitual control of behavior (Dolan & Dayan, 2013; Gillan & Robbins, 2014; Gillan et al., 2016; Robbins et al., 2019). Goal‐directed behaviors reflect knowledge of the relationship between an action (or sequence of actions) and outcome, and are performed only when the outcome is desirable or motivationally relevant at the moment of choice. In contrast, habits develop via previous reinforcement and are independent of the current value of the associated outcome. Characteristics of habitual control include automaticity, computational efficiency, and inflexibility, whereas goal‐directed control includes active deliberation, high computational cost, and flexibility in the face of changing environmental contingencies. For optimal functioning, the brain must carefully arbitrate between these two mechanisms. A number of psychiatric conditions have been associated with an imbalance between goal‐directed and habitual control, including addiction and obsessive‐compulsive disorder (OCD) (Gillan et al., 2016). A meta‐analysis found that one of the emotion dysregulation subscales that most strongly associated with NSSI was difficulties in engaging goal‐directed behavior (Wolff et al., 2019).

The aim of the present study was to investigate the dysfunction of goal‐directed control in patients with depression and NSSI (D‐NSSI). We used the Pavlovian‐to‐Instrumental Transfer (PIT) paradigm that assesses the effect of Pavlovian conditioned stimuli (CSs) on instrumental behavior. The canonical PIT paradigm begins with separate instrumental conditioning and Pavlovian conditioning, and then examines how inclusion of Pavlovian cues affects instrumental responding (Cartoni et al., 2016). Pavlovian cues typically have synergistic effects in which cues associated with reward enhance choice and vigor during instrumental responding and cues associated with loss have opposite effects. Recently, researchers argued that the human PIT paradigm largely involves goal‐directed, controlled process: it is sensitive to the participant's awareness of the contingency between the two tasks (whereas habit is largely implicit) and is sensitive to outcome devaluation (whereas habitual responding is outcome devaluation insensitive) (Mahlberg et al., 2019). We hypothesized that goal‐directed control in D‐NSSI would be deficient as in other disorders that are characterized by compulsivity (e.g., drug addiction, OCD). In PIT task, we predicted that impaired goal‐directed control would result in a lower PIT effect: that is, a reduced effect of CSs on instrumental responses. If so, we would interpret these results as an inability of those with D‐NSSI to use information given by CSs to adjust instrumental response using goal‐directed control. In addition, we used moderation analysis to further examine the relationship between PIT and compulsivity within D‐NSSI.

## 2 METHODS

### 2.1 Participants

A total of 37 patients with D‐NSSI and 34 healthy controls (HC) with normal or corrected to normal vision were recruited to participate in the study. All the participants were recruited within the People's Republic of China and were of Asian race and Han ethnicity. Two participants with D‐NSSI were excluded from further analysis because they failed to complete the experiment. D‐NSSI and HC were matched in sex and education but were dissimilar in age (*t*_40.687 _= −4.061, *p* < .001; see Table 1). Consequently, all data analyses were controlled for age. Patients were recruited from Shenzhen Kangning Hospital via referral from psychiatric medical personnel. HC were recruited via online advertising. Patients with D‐NSSI were diagnosed by a fully certified consultant psychiatrist according to DSM‐5 criteria and the results of the Mini‐International Neuropsychiatric Interview (Sheehan et al., 1998). If a patient with depression reported a history of NSSI behaviors, the psychiatrist used a questionnaire addressing 12 NSSI behaviors to assess the frequency of NSSI. Each of these behaviors was asked individually. For each behavior, patients reported the total number of times they had participated in that behavior during the previous 12 months. Overall, a total frequency of NSSI behaviors during the preceding year was calculated by summing these frequency scores across all the 12 NSSI items (You et al., 2012). This NSSI frequency score was used as a proxy for NSSI severity. All participants completed the Yale–Brown Obsessive‐Compulsive Scale (Y‐BOCS) (Goodman et al., 1989), the Beck Depression Inventory (BDI) (Beck & Steer, 1984), and the State‐Trait Anxiety Inventory (STAI) (Shahid et al., 2012) to assess severity of obsessive‐compulsive symptoms, depressive symptoms, and anxiety symptoms, respectively.

**TABLE 1:** Demographic and clinical characteristics

Characteristic	D‐NSSI (*n* = 35) mean (SD)	HC (*n* = 34) mean (SD)	*χ* ^2^/*t*	*p*
Demographic				
Female (%)	68.57	64.71	0.116^a^	.733
Age	26.14 (7.35)	20.85 (2.28)	4.06^b^	<.001
Education	14.23 (2.62)	14.68 (1.74)	0.84^b^	.41
Clinical				
Y‐BOCS obsessions	7.75 (4.81)	4.96 (3.29)	2.736^b^	.008
Y‐BOCS compulsions	5.59 (4.79)	3.12 (3.19)	2.454^b^	.017
Y‐BOCS total	13.34 (8.49)	8.07 (5.51)	2.971^b^	.004
STAI‐State	54.69 (13.79)	38.56 (10.38)	5.389^b^	<.001
STAI‐Trait	58.66 (10.77)	43.03 (9.33)	6.309^b^	<.001
STAI‐Total	113.34 (23.11)	81.59 (19.26)	6.078^b^	<.001
BDI	30.5 (12.83)	8.91 (6.55)	8.53^b^	<.001
D‐NSSI				
NSSI frequency^c^	20.72 (12.14)	0 (0)		

Abbreviations: BDI, Beck Depression Inventory‐II; D‐NSSI, depressed patients with nonsuicidal self‐injury; HC, healthy controls; STAI, State–Trait Anxiety Inventory; Y‐BOCS, Yale–Brown Obsessive‐Compulsive Scale.

^a^ Chi‐square tests.

^b^ Independent samples *t*‐tests.

^c^ Total number of behaviors reported on the questionnaire used to estimate NSSI frequency in the previous year. See text for details.

Exclusion criteria for both D‐NSSI and HC included patients being younger than 18 or older than 50, past or current neurological disorder, substance abuse/dependence, and endocrine or cardiac disorders. Furthermore, HC were excluded from the study if they had a past or current psychiatric disease or a family history of any psychiatric diseases in DSM‐V. Patients with D‐NSSI were excluded if they were diagnosed with psychiatric disorders other than depression and OCD and if they did not report a history of NSSI. One of the patients with D‐NSSI was also diagnosed with OCD.

The study was approved by the Human Research Ethics Committee for Clinical Faculties of Shenzhen Kangning Hospital. All methods were performed according to their relevant regulations and guidelines. All participants provided written informed consent for the study and received basic compensation of 30 RMB and a bonus based on task performance ranging from 0 to 5 RMB.

### 2.2 Procedure

Each participant completed the PIT paradigm (Garbusow et al., 2014) following this order: instrumental training (stage 1), Pavlovian training (stage 2), PIT (stage 3), and forced choice task (stage 4).

#### 2.2.1 Instrumental Training

Participants were required to collect shells to obtain as many coins as possible. Instrumental stimuli comprised two “good shells” (“G” and individually as “G1” and “G2”) and two “bad shells” (“B” and individually as “B1” and “B2”). To collect G, participants were required to repeatedly press the “Enter” button to move the red dot to the center of the G. To avoid the collection of B, participants had to refrain from pressing the “Enter” button enough times for the red dot to enter the center of the B. Collecting G or not collecting B was rewarded on 80% and punished on 20% of trials. Collecting B or not collecting G was rewarded on 20% and punished on 80% of trials. Participants were under time pressure to respond rapidly due to the limited response window. They were not informed as to how many button presses were required, which also resulted in pressure to respond rapidly.

**FIGURE 1:**
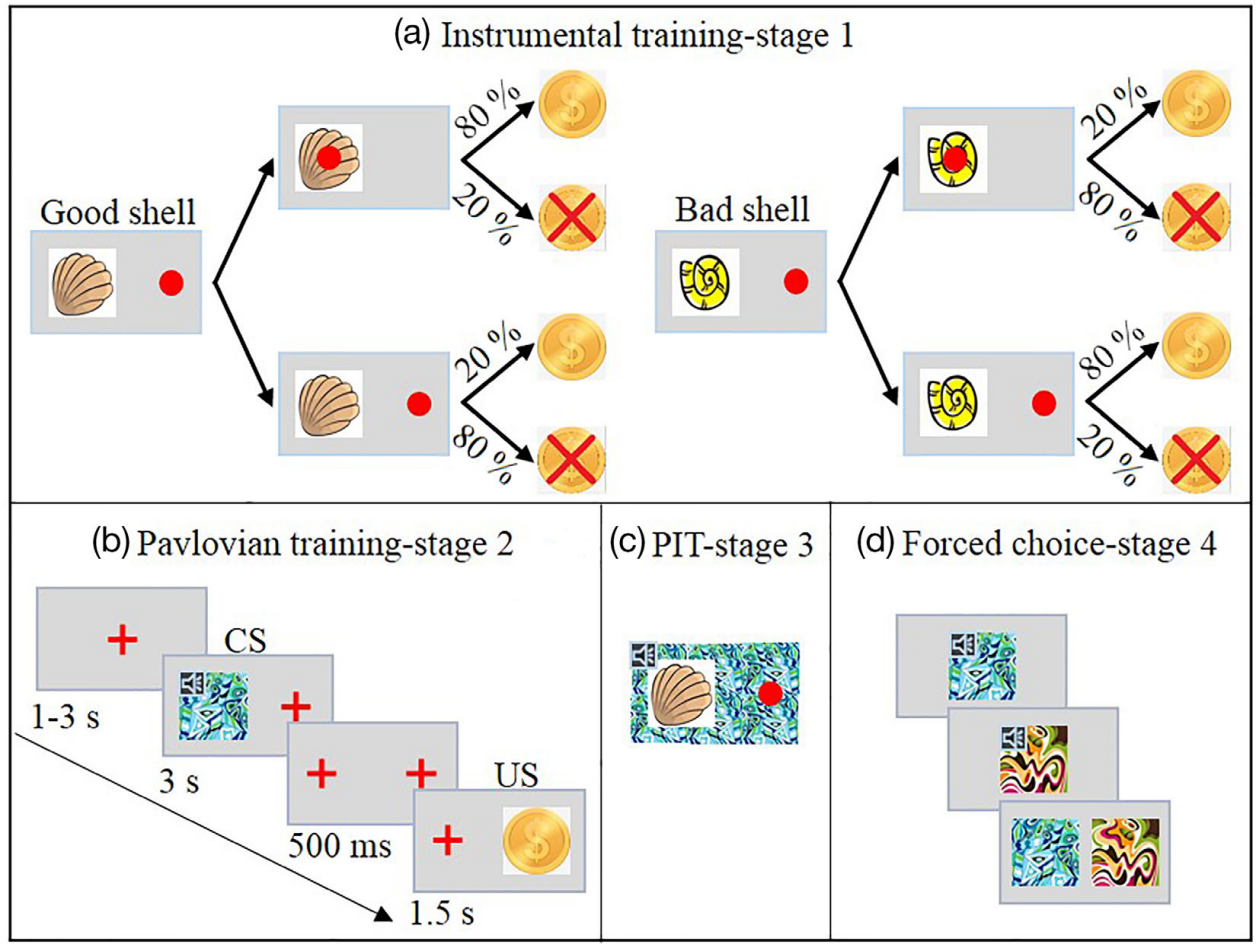
Pavlovian‐to‐Instrumental Transfer (PIT) paradigm. (a) Instrumental training. Participants learned to classify good shells or bad shells and probabilistically gain coins as a reward. Participants responded by pressing the Enter key multiple times; each press moved the red dot incrementally closer to the center of the shell. Participants learned to collect the good shells to gain coins (80% probability of gain if collected), and refrain from collecting the bad shells to gain coins (80% probability of gain if the shell was not collected). (b) Pavlovian training. Participants were asked to learn the conditioned stimulus (CS)‐unconditioned stimulus (US) pairings. (c) PIT. This phase was design to test the influence of CSs on instrumental conditioning. Participants performed the same task as in (a), but with simultaneous presentation of a CS, without explicit indication of reward obtained. (d) Forced choice task. Participants viewed pairs of CSs and indicated which one of the pair had the highest value

Instrumental training started with a red fixation cross (+) presented for a random interval ranging between 1 and 3s. Then, a shell appeared on the right or left side of the screen (counterbalanced), and participants had 2 s to repeatedly press or avoid pressing the “Enter” key to collect or not collect the shell. There were 80 trials divided into three blocks of unequal lengths. The first two blocks included only two of the stimuli, G1 and B1 on block 1, and G2 and B2 on block 2, and lasted for 20 trials each. This design enabled participants to focus on learning just two stimuli at a time and was developed after pilot testing indicated that participants found it difficult to learn all four stimuli simultaneously. Block 3 included all four stimuli and lasted for a maximum of 40 trials. To ensure that all participants learned they were not considered trained until they completed 80% correct choices over 10 consecutive trials and completed a minimum of 10 trials in the last block (Figure 1a).

#### 2.2.2 Pavlovian training

Participants were instructed to carefully observe and memorize the conditioned stimuli (CS) ‐ unconditioned stimulus (US) associations and completed 24 trials (Figure 1b). Specifically, stage 2 began with presentation of a red fixation cross followed by a conditioned stimulus. The CS were each a combination of an abstract visual image along with a series of auditory pure tones. A total of three CSs were presented and were randomly assigned to CS+, CS0, and CS‐ roles across participants. The visual portion of each CS was randomly presented on the right or left side of the screen for 3 s, while the auditory component was played over the computer headphones. The CS was followed by a 500 ms interval and then the US was presented on the opposite side of the screen for 1.5 s. Overall, there were three CS‐US pairings: one positive CS (+) paired with a positive US (a coin), one neutral CS (0) paired with a neutral US (a blank), and one negative CS (−) paired with a negative US (a coin with a superimposed red cross). The CS‐US pairings were deterministic and counterbalanced between and within participants.

#### 2.2.3 Pavlovian‐to‐Instrumental transfer

Participants were told that this phase was similar to the instrumental training and they should try their best to obtain coins again. This stage was identical to the instrumental training with the following exceptions: First, while the shells were presented, task‐irrelevant CSs (from stage 2, including both the visual and auditory components) appeared as a background, and second, the whole task was completed in nominal extinction without feedback being displayed in order to avoid any confounding effects of new learning. The task consisted of 72 trials, with each stimulus‐CS combination appearing 12 times (Figure 1c).

#### 2.2.4 Forced choice task

Stage 4 (Figure 1d) was used to verify that participants had acquired the CS‐US associations. CSs pairs were presented and participants were instructed to choose the best CS (most valuable) of the two. All possible combinations of two CSs were presented in an interleaved, randomized order three times. If participants failed to perform better than chance, they were excluded from further analyses.

## 3 RESULTS

All data analyses were controlled for age. The analyses were also repeated with the D‐NSSI patient with OCD excluded; this exclusion had little effect on our results.

### 3.1 Instrumental training

**FIGURE 2:**
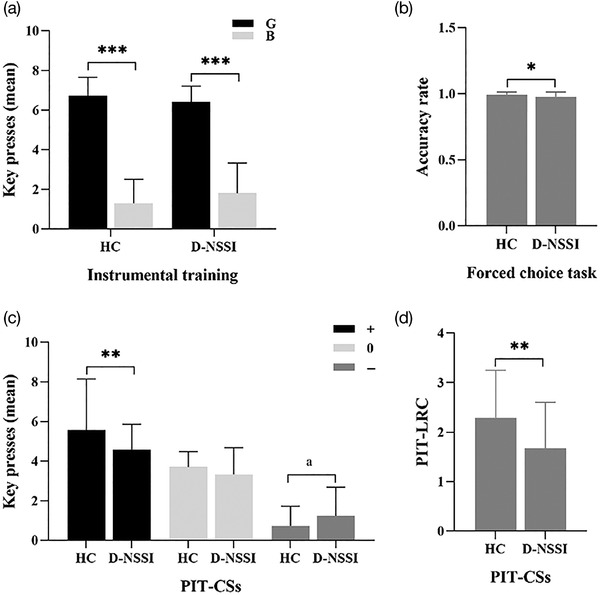
Pavlovian‐to‐Instrumental Transfer (PIT) performance. (a) Mean key presses for good stimuli (G) and bad stimuli (B) during instrumental training. (b) Accuracy rate (proportion correct) for the forced choice task. (c) Mean number of key presses for each conditioned stimulus (CS). (d) The total PIT effect (PIT‐LRC, PIT‐linear regression coefficients). G: good shells (stimuli associated with potential gain). B: bad shells (stimuli associated with potential loss). CS: conditioned Stimulus. +: CS+. 0: CS0. −: CS−. “*”: *p *< .05, “**” *p *< .01, “***”: *p *< .001, “a”: *p *= .068

Mean number of trials (across all blocks) that participants needed to accomplish this learning were 50.0 and 50.8 in HC and D‐NSSI groups, respectively, and did not differ between groups (Mann–Whitney *U* Test: *p *= .083). The total number of trials was very close to the minimum required (total of 20 each in blocks 1 and 2, and a minimum of 10 trials in block 3). Repeated measures analysis of variance (ANOVA) was used for the mean key presses, including stimulus type and participant group as factors. There was a main effect of stimuli, with higher mean key presses for G than B shells (*F*_1,66 _= 32.28, *p *< .001). There was no effect of group (*F*_1,66 _= 0.02, *p *= .891) and no interaction between stimuli × group (*F*_1,66 _= 2.058, *p *= .156), indicating that instrumental conditioning in both groups was similar. To ensure that both groups learned the instrumental conditioning, we performed paired‐samples *t*‐tests on mean key presses for G and B for the D‐NSSI and HC groups separately. Participants learned to press more often when collecting was rewarded (G) and to press less when terminating was rewarded (B) in both groups (D‐NSSI: *t*_34 _= 14.923, *p *< .001; HC: *t*_33 _= 18.132, *p *< .001, Figure 2a).

### 3.2 Pavlovian training

Individual acquisition of Pavlovian associations was assessed by accuracy rate in the forced choice task. Group comparisons were performed with Mann–Whitney *U* tests. All participants preferred higher valued CSs overall, and no participants were eliminated due to failure to meet the criterion of above‐chance performance. The mean accuracy rates of D‐NSSI and HC were both very high, 0.98 and 0.99, respectively. The small numerical difference in accuracy reached statistical significance (*p *= .024, see Figure 2b).

### 3.3 Pavlovian‐to‐Instrumental Transfer

Repeated measures ANOVA was used with stimuli (G and B) and CSs (CS+, CS0, and CS− ) as within‐participants factors and group (HC and D‐NSSI) as a between‐participants factor. The mean key presses for each stimulus‐CS combination were the dependent variable. We found a significant interaction of group × stimuli × CSs (*F*_1.72,113.66 _= 4.53, *p *= .017), group × CSs (*F*_1.72,113.32 _= 7.20, *p *= .002), and stimuli × CSs (*F*_1.72,113.66 _= 7.11, *p *= .002). In addition, there were significant main effects of stimuli (*F*_1,66 _= 51.83, *p *< .001) and CSs (*F*_1.72,113.32 _= 5.75, *p *= .006). The group × stimuli interaction (*F*_1,66 _= 1.81, *p *= 0.183) and main effect of group (*F*_1,66 _= 0.75, *p *= .388) were not significant.

We then tested differences between groups as a function of CS valence collapsed across stimulus type (Figure 2c). HC performed significantly more key presses in the CS+ than the D‐NSSI group (*F*_1,66 _= 8.19, *p *= .006). There was a trend toward fewer key presses in the CS‐in the HC group than in the D‐NSSI group (*F*_1,66 _= 3.44, *p *= .068). All of the comparisons were consistent with a lower effect of the CS on responding in the D‐NSSI group.

Finally, we assessed differences in sensitivity to CSs between groups via regression in which we regressed each participant's mean number of key presses (across all CSs) onto positive, neutral, and negative (+1, 0, −1) CSs (Huys et al., 2016). The HC had larger resulting linear regression coefficients (PIT‐LRC, a measure of each participant's overall PIT effect in the individual difference analyses) than the patients with D‐NSSI (*F*_1,66 _= 11.21, *p *= .001; Figure 2d), indicating a larger effect of CS on instrumental performance across all CS valences.

### 3.4 Behavioral and clinical relationships

In order to examine associations between PIT and clinical variables from the Y‐BOCS and NSSI frequency questionnaire, we performed correlation analyses (controlled for age, anxiety [STAI‐total], and depression [BDI]) on data limited to the D‐NSSI group. For the CS+ condition, there were significant negative correlations between mean key presses and YBOCS (total: *r *= −0.42, *p *= .026. obsessions: *r *= −0.47, *p *= .011), and a trend toward correlation with NSSI frequency (*r *= −0.36, *p *= .060). This correlation indicated that participants with higher NSSI frequency showed a lower effect of the CS+ on responding.

**FIGURE 3:**
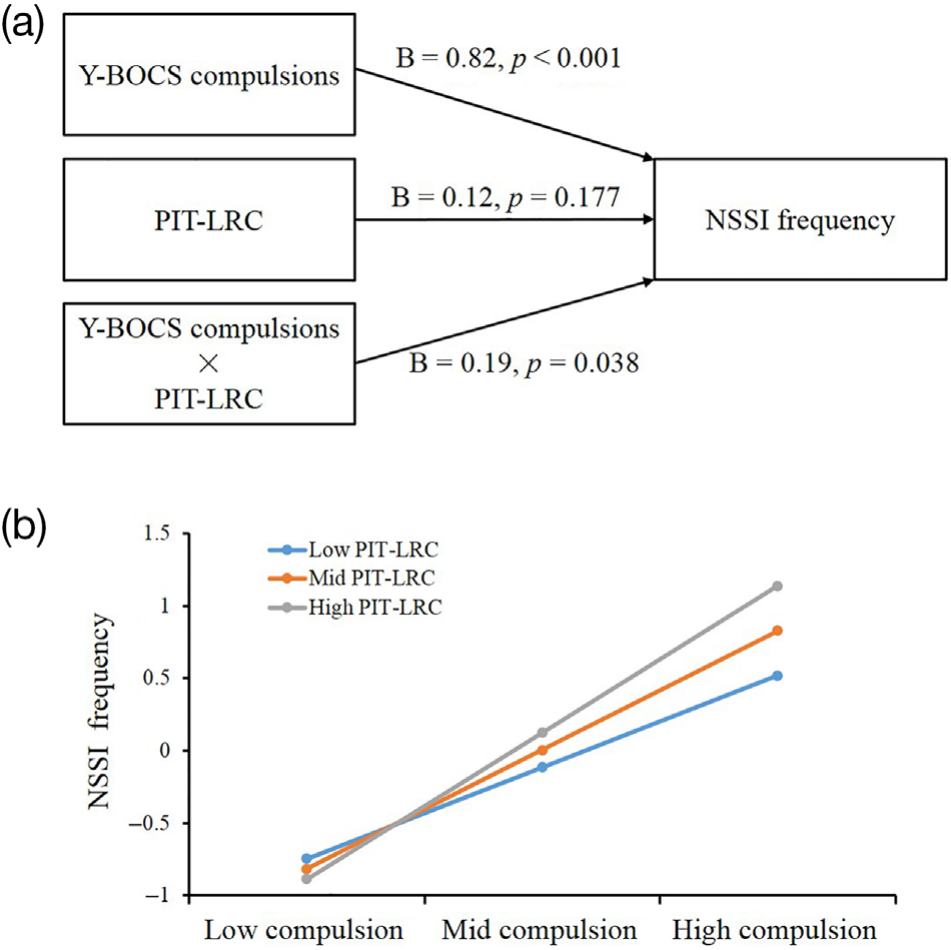
Moderation analysis. (a) Moderating effect of Pavlovian‐to‐Instrumental Transfer (PIT)‐linear regression coefficients (PIT‐LRC, a measure of each participant's overall PIT effect in the individual difference analyses) on the relation between Yale–Brown Obsessive‐Compulsive Scale (Y‐BOCS) compulsions and nonsuicidal self‐injury (NSSI) frequency. (b) The relation between Y‐BOCS compulsions and NSSI frequency in low, average, and high levels of Y‐BOCS compulsions and PIT‐LRC (one SD below the mean, mean, and one SD above the mean, respectively). Abbreviations: D‐NSSI, depressed patients with NSSI; HC, healthy controls

We used moderation analysis to examine how goal‐directed control as measured using PIT (PIT‐LRC) affects the relationship between compulsivity and NSSI frequency (SPSS process v3.3 analysis package, http://processmacro.org/index.html). NSSI frequency was entered as output variable, Y‐BOCS compulsions as x variable, and PIT‐LRC as moderator variable. Age, STAI‐total, and BDI were entered as covariates. All data were standardized using z‐scores. There was a significant positive relationship between Y‐BOCS compulsions and NSSI frequency (*B* = 0.82, *p *< .001, Figure 3a), and this effect was positively moderated by the PIT‐LRC (*B* = 0.19, *p *= .038). Furthermore, the interaction between compulsions and PIT‐LRC was apparent primarily at high levels of compulsions, with PIT‐LRC predicting greater NSSI frequency above that accounted for by compulsion alone. Little effect was apparent for participants with low or intermediate levels of compulsion. Simple slope tests revealed significant simple moderation effects for low and high levels of PIT‐LRC (low: *B* = 0.63, SE = 0.14, *t *= 4.45, *p *< .001; high: *B* = 1.01, SE = 0.13, *t *= 7.62, *p *< .001; Figure 3b).

## 4 DISCUSSION

The present study examined goal‐directed control in patients with D‐NSSI using the PIT paradigm. We found the D‐NSSI group showed lower total PIT effects than HC when measured by the PIT‐LRC variable and when examining individual CSs in isolation. These findings support the theory that D‐NSSI is associated with dysfunction of goal‐directed control, with reduced use of the information provided by the different CSs to control ongoing goal‐directed behavior. Furthermore, an intriguing interaction between the Y‐BOCS compulsions measure and PIT was revealed. Specifically, our moderation analysis found that PIT‐LRC levels positively moderated the effect of compulsivity on NSSI frequency, such that those with both high compulsivity and high PIT had particularly high frequency of NSSI. Notably, this pattern was found only in participants with high degrees of compulsivity. It is consistent with previous studies of high compulsivity populations, including addiction, in which PIT was shown to be positively related to compulsivity and was also a predictor of future alcohol use and relapse in alcohol use disorder (Sekutowicz et al., 2019; Sommer et al., 2017).

In line with previous studies, we supported the view that specific PIT effects in humans are supported by goal‐directed behavioral control functions. One important source of evidence comes from outcome devaluation procedures in which rewards are removed or reduced in value; goal‐directed behavior is reduced after outcome devaluation, whereas habitual behavior is unaffected. PIT is reduced by outcome devaluation, which suggests that PIT reflects a goal‐directed, controlled process in humans (Mahlberg et al., 2019; Seabrooke et al., 2017). According to the propositional theory, participants learn response‐outcome (R‐O) and stimulus‐outcome (S‐O) relationships during instrumental training and Pavlovian training stages, respectively. These learned associations then support goal‐directed control in the transfer phase so that participants are able to flexibly use CSs to infer which outcomes are available and which responses will be effective. Our result found overall impairment in D‐NSSI in the ability to flexibly use the information from CSs to control ongoing instrumental responses.

The present results revealed that patients with D‐NSSI were less able to use information about potential reward availability (as signaled by the CSs) to enhance goal‐directed behavior. Other studies of NSSI have found similar impairment in using contextual cues in behavioral control. Patients with NSSI manifested poorer inhibition to images with negative emotional content on a Stop Signal Task (Allen & Hooley, 2015).

Studies have also found impairment in reward processing in NSSI. One study found greater correlation between activity in the dorsal striatum and reward (implemented as relief from pain) in patients with NSSI than in control patients without NSSI (Osuch et al., 2014). In addition, individuals with Borderline Personality Disorder comorbid with NSSI exhibited OFC overactivation following an unexpected reward in a gambling task, when compared with controls. OFC overactivation has been proposed as a possible phenotype for reward‐related alterations in NSSI (Vega et al., 2018).

Previous studies have also found different patterns of activity in brain regions associated with goal‐directed and habitual control in NSSI than in controls, with higher activity in neural regions associated with habitual learning. Adolescents who engaged in present or past NSSI thoughts without NSSI behavioral history showed enhanced putamen activation (typically associated with habitual processing) in response to a monetary reward (Poon et al., 2019).

It is important to note that we found a relationship between compulsivity and NSSI frequency, with higher levels of compulsivity related to greater frequency of NSSI. Moreover, the interaction between compulsions and PIT‐LRC was apparent primarily at high levels of compulsions, with PIT‐LRC predicting greater NSSI frequency than accounted for by compulsion alone. To the best of our knowledge, our study was the first to report empirical evidence supporting a role for compulsivity in NSSI. An imbalance between goal‐directed and habitual control is a typical characteristic of disorders characterized by compulsivity (Gillan et al., 2016), including drug addiction, binge eating disorders, and OCD, with compulsivity typically related to higher effects of CSs on behavior. The current PIT results demonstrated that there was an overall deficit of the capacity to use Pavlovian cues to flexibly adjust ongoing instrumental behaviors in patients with D‐NSSI. Furthermore, the findings of relationships between compulsivity, PIT, and NSSI frequency may have clinical significance. Because the severity of obsessive‐compulsive symptoms may have impacts on NSSI, a separate treatment plan should be adopted in clinical treatment according to the severity of obsessive‐compulsive symptoms in patients with NSSI.

This study has several limitations of note. First, the current study used a cross‐sectional design making it difficult to determine whether the imbalance between the two control systems is a cause or a result of NSSI. Future studies using longitudinal designs would help to understand this imbalance. Second, patients in this study were also diagnosed with depression. Although the co‐occurrence of depression and NSSI is very common (Bentley et al., 2014; Jacobson & Gould, 2007; Nock et al., 2006), future studies should recruit another group of depressed patients without NSSI to perform the same task in order to assess the relative influence of NSSI and depression on goal‐directed learning. Third, all of the participants were Asian and of Han ethnicity and were citizens and residents of the People's Republic of China. Future research should examine participants from other nations, races, and ethnicities to assess the generality of the results to other groups. Finally, future study should focus on a better understanding of NSSI beyond depression. Although patients with NSSI who do not have depression do exist, it is rare and is a difficult‐to‐recruit group. We would expand the sample size to include most other comorbid disorders, in order to collecting a more comprehensive and representative sample of patients with NSSI.

In conclusion, we found impaired goal‐directed control in D‐NSSI related to the inability to use contextual cues to affect instrumental behavior. Therapeutic research may focus on interventions to enhance the capacity of using environmental information to help those with NSSI to control their ongoing behavior. We also found a positive relationship between compulsivity, PIT, and NSSI frequency which was greatest for high compulsivity patients with D‐NSSI. This heterogeneity within D‐NSSI group suggests that patients with NSSI with low and high compulsivity may require and benefit from different therapeutic interventions.

## CONFLICT OF INTEREST

The authors declare no conflict of interest.

### PEER REVIEW

The peer review history for this article is available at https://publons.com/publon/10.1002/brb3.2607.

## Data Availability

The data presented in this study are available on request from the corresponding author. The data are not publicly available due to privacy reasons.
